# *Cdkl5* Knockout Mice Recapitulate Sleep Phenotypes of CDKL5 Deficient Disorder

**DOI:** 10.3390/ijms26083754

**Published:** 2025-04-16

**Authors:** Liqin Cao, Xin Zhang, Tingting Lou, Jing Ma, Zhiqiang Wang, Staci J. Kim, Kaspar Vogt, Arisa Hirano, Teruyuki Tanaka, Yoshiaki Kikkawa, Masashi Yanagisawa, Qinghua Liu

**Affiliations:** 1 International Institute for Integrative Sleep Medicine (WPI-IIIS), University of Tsukuba, Tsukuba 305-8575, Japan; zhangxin.luby@gmail.com (X.Z.); louting314@163.com (T.L.); jingmawzq@foxmail.com (J.M.); zhiqiang.wangmj@foxmail.com (Z.W.); staci.jkim@kaist.ac.kr (S.J.K.); vogt.kaspar.fu@u.tsukuba.ac.jp (K.V.); hirano.arisa.gt@u.tsukuba.ac.jp (A.H.); yanagisawa.masa.fu@u.tsukuba.ac.jp (M.Y.); 2 Deafness Project, Department of Basic Medical Sciences, Tokyo Metropolitan Institute of Medical Science, Tokyo 156-8506, Japan; kikkawa-ys@igakuken.or.jp; 3 HIT Center for Life Sciences (HCLS), School of Life Sciences and Technology, Harbin Institute of Technology, Harbin 150001, China; 4 Department of Brain and Cognitive Sciences, Korea Advanced Institute of Science & Technology, Daejeon 34141, Republic of Korea; 5 Tokyo Children Rehabilitation Hospital, Tokyo 208-0011, Japan; tetanaka@m.u-tokyo.ac.jp; 6 Department of Developmental Medical Sciences, Graduate School of Medicine, The University of Tokyo, Tokyo 113-0033, Japan; 7 National Institute of Biological Sciences (NIBS), Beijing 102206, China; 8 Tsinghua Institute of Multidisciplinary Biomedical Research (TIMBR), Tsinghua University, Beijing 100084, China

**Keywords:** CDKL5, mouse models, sleep, electroencephalography (EEG), circuit function

## Abstract

Cyclin-dependent kinase-like 5 (CDKL5) deficiency disorder (CDD) is an X-linked rare neurodevelopmental disorder associated with severe sleep disturbances. However, little is known about the mechanisms underlying sleep disturbances in CDD patients. Here, we employed the electroencephalogram (EEG) recording to characterize sleep–wake behaviors and EEG activity in male CDKL5-deficient mice. We found that young adult and middle-aged *Cdkl5* knockout (KO) mice recapitulated sleep phenotypes in patients with CDD, including difficulties in initiating and maintaining sleep, reduction in total sleep time, and frequent night awakenings. *Cdkl5* KO mice exhibited pre-sleep arousal, but normal circadian rhythm and homeostatic sleep response. Conditional knockout (cKO) of *Cdkl5* in glutamatergic neurons resulted in reduced sleep time and difficulty in sleep maintenance. Further, the rate of age-associated decline in sleep and EEG activity in *Cdkl5* KO mice was comparable to that of wild-type littermates. Together, these results confirm a causative role for CDKL5 deficiency in sleep disturbances observed in CDD patients and establish an animal model for translational research of sleep treatment in CDD. Moreover, our results provide valuable information for developing therapeutic strategies and identifying sleep and EEG parameters as potential biomarkers for facilitating preclinical and clinical trials in CDD.

## 1. Introduction

Cyclin-dependent kinase-like 5 (CDKL5) deficiency disorder (CDD) is a rare neurodevelopmental disorder caused by mutations in the X-linked *CDKL5* gene. CDD is characterized by early-onset refractory seizures, severe intellectual disability, autistic features, and visual and motor impairments [1,2,3,4,5,6]. Most (~90%) CDD patients suffer severe sleep disturbances, mainly problems of initiating and maintaining sleep, frequent night awakenings, and daytime sleepiness [7,8,9]. Notably, a longitudinal polysomnographic study of five girls with CDD reported long sleep latency, frequent night awakenings, shorter total sleep time, decreased rapid-eye-movement sleep (REMS), and low sleep efficiency, which persisted during the study period (5.5–10 years) [10,11]. Sleep disturbances worsen seizures, impair learning and memory [12,13,14], and have a significant negative impact on the quality of life of CDD patients and their caregivers [15,16]. However, relatively little is known about the mechanisms underlying sleep disturbances in CDD, and effective treatments for these sleep problems are not yet available.

Electroencephalogram (EEG) abnormalities have also been found in patients with CDD, which progressively deteriorated with the progression of epilepsy [17,18]. The band power of EEG signals is being increasingly incorporated as a noninvasive potential translatable biomarker of brain function, disease severity, and treatment response in many disorders, including neurodevelopmental disorders [19,20,21]. Reliable and sensitive biomarkers to assess the clinical severity and the efficacy of novel therapies are essential for the success of clinical trials in CDD, which are currently lacking. In the neurodevelopmental disorders Rett syndrome (RTT) and Angelman syndrome (AS), enhanced delta (1–4 Hz) power in sleep were reported in patients. The strength of delta power negatively correlated with clinical severity across multiple domains including sleep efficiency, cognition, motor, and communication function [22,23,24,25], suggesting that delta power of sleep EEG may be useful as a potential biomarker in RTT and AS. To date, only two studies analyzed the resting EEG in a small number of CDD patients [26,27], and quantitative analysis using the sleep EEG has not been reported.

To fully understand the mechanisms underlying sleep disturbances and explore the potential utility of sleep and quantitative EEG parameters as functional and therapeutic biomarkers in CDD, animal models that closely recapitulate sleep phenotypes observed in CDD patients are needed, given that in CDD patients, several factors, including intrinsic CDKL5 loss-of-function pathology, secondary impact of seizures, and medication, are implicated in sleep problems and EEG signal alterations [28,29]. Mouse models of CDD that mimic primary symptoms of CDD patients have been developed [30,31,32,33,34]. However, only one study has examined the daily sleep amount in *Cdkl5* knockout (KO) mice using non-invasive whole-body plethysmography [35]. In the present study, we characterized the sleep phenotypes and EEG power spectra of young and middle-aged *Cdkl5* KO and conditional *Cdkl5* KO mice by electroencephalogram (EEG)/electromyogram (EMG) recording. We found that *Cdkl5* KO mice recapitulate sleep phenotypes of CDD patients and exhibit similar altered EEG activity across ages. Loss of CDKL5 in excitatory neurons results in reduced sleep amount and sleep fragmentation. Our findings suggest that *Cdkl5 KO* mice are an excellent animal model for studying sleep disturbances of CDD patients, and sleep and EEG parameters have potential as translational biomarkers for CDD.

## 2. Results

### 2.1. Cdkl5 KO Mice Recapitulate Sleep Disturbances Observed in CDD Patients

To understand the natural history of sleep in *Cdkl5* KO mice, we performed sleep analysis in young adult (3-month-old, hereon referred as young) and middle-aged (12-month-old, hereon referred to as aged) *Cdkl5^-/Y^* and their littermate control *Cdkl5^+/Y^* mice (hereon referred to as KO and WT, respectively). Given the confounding effects of mosaic CDKL5 expression in females due to random X-chromosome inactivation, we used only male mice in this study.

**Figure 1:**
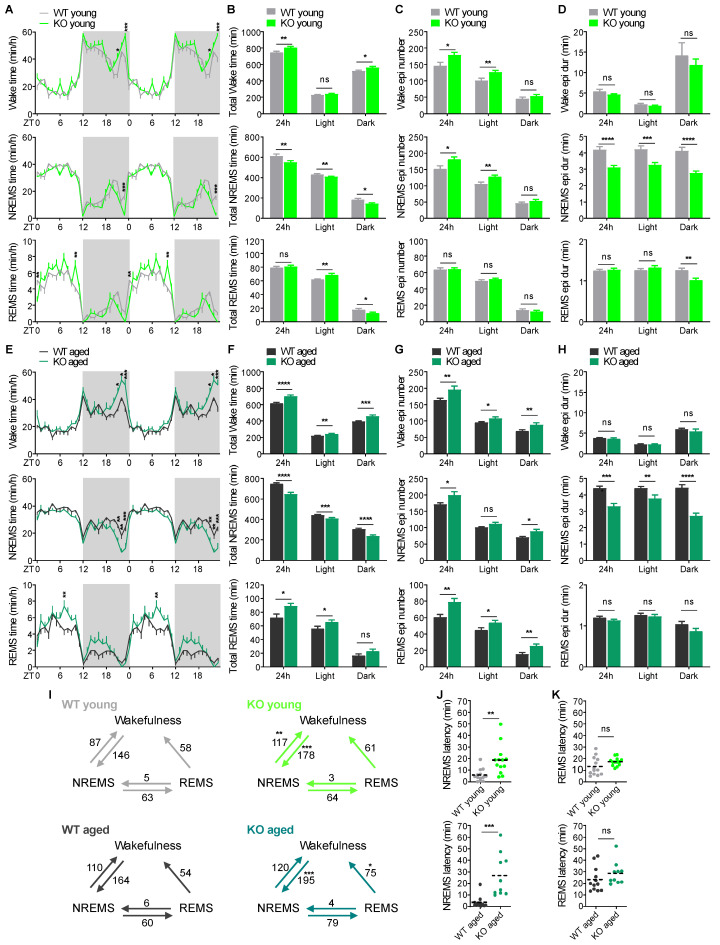
*Cdkl5* KO mice recapitulate sleep disturbances observed in CDD patients. (**A**–**D**) Hourly time (**A**), total time (**B**), numbers of episodes (**C**), and mean durations of episodes (**D**) of wakefulness, NREMS, and REMS in young WT (*n* = 13) and *Cdkl5* KO mice (*n* = 13). Time points are double-plotted to facilitate visual detection of daily variation. ZT, Zeitgeber time. (**E**–**H**) Hourly time (**E**), total time (**F**), numbers of episodes (**G**), and mean durations of episodes (**H**) of wakefulness, NREMS, and REMS in aged WT (*n* = 12) and *Cdkl5* KO mice (*n* = 10). Time points are double-plotted to facilitate visual detection of daily variation. (**I**) Number of transitions between wakefulness, NREMS, and REMS per 24 h in young (up) and aged (down) WT and KO mice. Arrows show the direction of transitions, and numbers show the average frequency of transitions. (**J**) Mean NREMS latency in young (up) and aged (down) WT and KO mice. (**K**) Mean REMS latency in young (up) and aged (down) WT and KO mice. Data are the mean ± SEM. Two-way repeated measures ANOVA with Sidak’s test (**A**,**E**,**I**) and unpaired *t*-test (**B**–**D**,**F**–**H**,**J**,**K**). * *p* < 0.05; ** *p* < 0.01; *** *p* < 0.001; **** *p* < 0.0001. ns, not significant.

No spontaneous seizures were observed in our young and aged KO mice through video-EEG recording for at least 96 h. KO mice exhibited comparable EEG/EMG signals relative to WT littermates (Figure S1A). Over the 24 h baseline recording, both young and aged KO mice showed similar diurnal sleep–wake rhythms compared to WT littermates (Figure 1A,E). KO mice spent more time awake and less time in non-rapid eye movement sleep (NREMS) than WT littermates at both ages. REMS of young KO mice was comparable to that of WT mice, whereas REMS in aged KO mice was significantly enhanced, suggesting an age-dependent increase in REMS in KO mice (Figure 1B,F).

To determine in which hours these effects were most pronounced, we examined hourly time for wakefulness, NREMS, and REMS over 24 h using two-way repeated-measures ANOVA. Interestingly, there was a significant increase in wakefulness and a concomitant decrease in NREMS at zeitgeber time 23 (ZT23) in young KO mice and at ZT21-23 in aged KO mice, respectively (Figure 1A and Figure 1E). These data suggest that KO mice experienced pre-sleep arousal at both ages, and the duration of pre-sleep arousal was further increased in aged KO mice. The proportions of sleep (combined time in NREMS and REMS) and wake across 6 h blocks over 24 h were similar between genotypes in young animals, suggesting that the reduction of sleep in young KO mice was constant across the 24 h cycle rather than in a particular time period (Figure S1B,C). The same is true for aged KO mice except for a decreased proportion of sleep in the last half of the dark phase in parallel with the enhanced pre-sleep arousal in aged KO mice (Figure S1D,E).

KO mice showed a more fragmented sleep–wake cycle compared to WT littermates at both ages. Specifically, young and aged KO mice had a significant increase in the overall episode number of both wake and NREMS (Figure 1C,G and Figure S1F,G), and shorter mean episode duration of NREMS (Figure 1D,H and Figure S1H,I). Aged KO mice also had more episodes of REMS (Figure 1G and Figure S1G) without an impact on their episode duration (Figure 1H and Figure S1I). Consistently, KO mice switched more often between sleep and wake states than WT littermates. There was a significant increase in the number of wake–NREMS and NREMS–wake transitions in young KO mice and wake–NREMS and REMS–wake transitions in aged KO mice (Figure 1I). KO mice exhibited a longer latency to NREMS after the onset of the light phase at both ages compared to WT littermates (Figure 1J), indicating a difficulty in initiating sleep. There was no significant difference in latency to REMS between genotypes (Figure 1K).

### 2.2. Cdkl5 KO Mice Exhibit Altered Baseline EEG Activity

**Figure 2:**
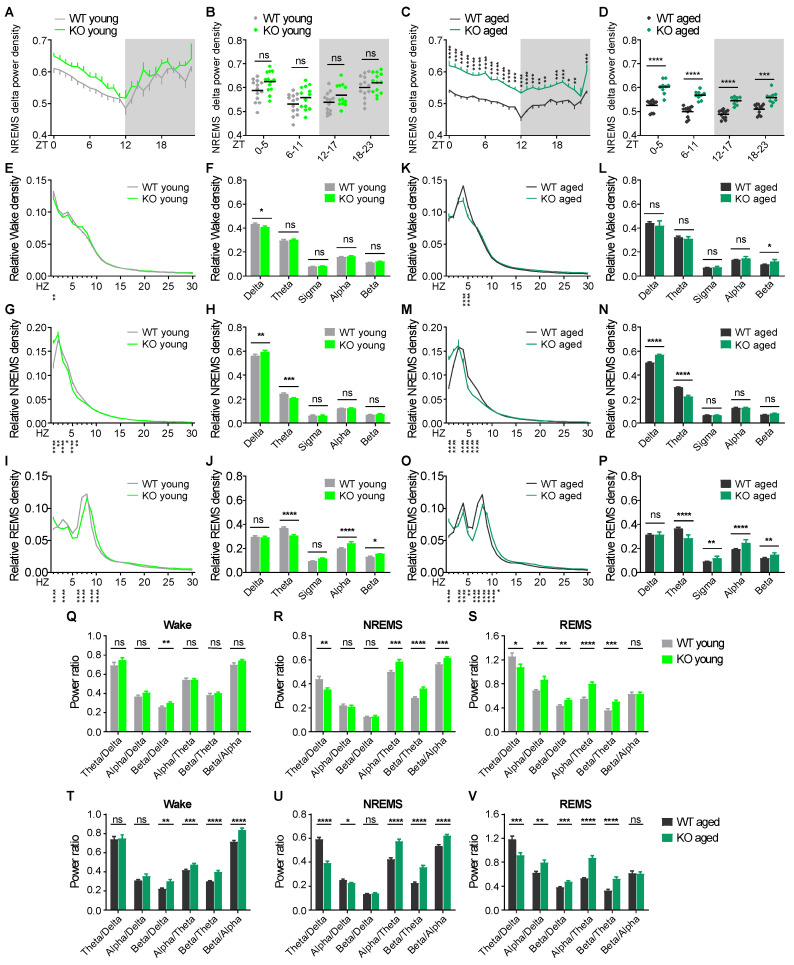
*Cdkl5* KO mice exhibit altered baseline EEG activity. (**A**,**B**) Mean NREMS delta power in every hour (**A**) and per 6 h block (**B**) in young WT (*n* = 13) and *Cdkl5* KO mice (*n* = 13). (**C**,**D**) Mean NREMS delta power in every hour (**C**) and per 6 h block (**D**) in aged WT (*n* = 11) and *Cdkl5* KO mice (*n* = 9). (**E**–**J**) EEG power spectra and frequency bands during wakefulness (**E**,**F**), NREMS (**G**,**H**), and REMS (**I**,**J**) in young WT and KO mice. (**K**–**P**) EEG power spectra and frequency bands during wakefulness (**K**,**L**), NREMS (**M**,**N**), and REMS (**O**,**P**) in aged WT and KO mice. (**Q**–**V**) EEG frequency band ratios during wakefulness, NREMS, and REMS in young (**Q**–**S**) and aged (**T**–**V**) WT and KO mice. Data are the mean ± SEM. Two-way repeated measures ANOVA with Sidak’s test (**A**–**P**) and unpaired *t*-test (**Q**–**V**). * *p* < 0.05; ** *p* < 0.01; *** *p* < 0.001; **** *p* < 0.0001. ns, not significant.

We first examined the diurnal pattern of NREMS delta (1–4 Hz) power density, a well-established electrophysiological marker of sleep need and a measure of synchrony of the neural network [36]. KO mice exhibited constitutively elevated NREMS delta power across the 24 h period at both ages (Figure 2A,D). These results suggest that KO mice do not have an intrinsic decreased sleep need, and the neural activity of KO mice in NREMS is hyper-synchronized. Next, we analyzed the EEG power spectra of KO and WT mice to detect difference across a wide range of frequencies (Figure 2E,P). During wakefulness, young KO mice exhibited decreased delta power, whereas age KO mice exhibited increased beta power and tended to have a reduction in delta power compared to WT littermates (*p* = 0.069) (Figure 2F,L). During NREMS, KO mice showed an increase in delta power and decrease in theta power (5–8 Hz) compared to WT littermates at both ages. The power in the sigma band (10–12 Hz), which corresponds to the frequency of sleep spindles in mice [37], was similar between genotypes (Figure 2H,N). During REMS, EEG power spectra of KO mice were shifted toward higher frequencies at both ages (Figure 2I,O). KO mice had decreased power in theta, and increased power in alpha (9–14 Hz) and beta bands (15–30 Hz). Additionally, aged KO mice showed increased power in sigma band (Figure 2J,P). Similar changes in the EEG spectra of KO mice were observed in both light and dark phases (Figure S2).

EEG power frequency band ratio measures, referring to the ratio of power between two frequency bands, are commonly used across basic and clinical neuroscience to seek biomarkers for diagnosis [38,39]. To this end, we calculated band ratio measures. During wakefulness, young and aged KO mice displayed a higher EEG beta/delta ratio. Further, aged KO mice exhibited higher alpha/theta, beta/theta, and beta/alpha ratios compared to their WT littermates, indicating an age-dependent increase in higher versus lower frequency band power (Figure 2Q,T). During NREMS, KO mice had lower theta/delta, higher alpha/theta, beta/theta, and beta/alpha ratios compared to WT littermates at both ages. Additionally, aged KO mice had a lower alpha/delta ratio than WT mice (Figure 2R,U). During REMS, young and aged KO mice showed a similar decrease in theta/delta and an increase in alpha/delta, beta/delta, alpha/theta, and beta/theta ratios compared to WT littermates of the same age (Figure 2S,V).

### 2.3. Loss of Cdkl5 Does Not Exacerbate the Rate of Age-Associated Changes in Sleep Behavior and EEG Spectra in Mice

**Figure 3:**
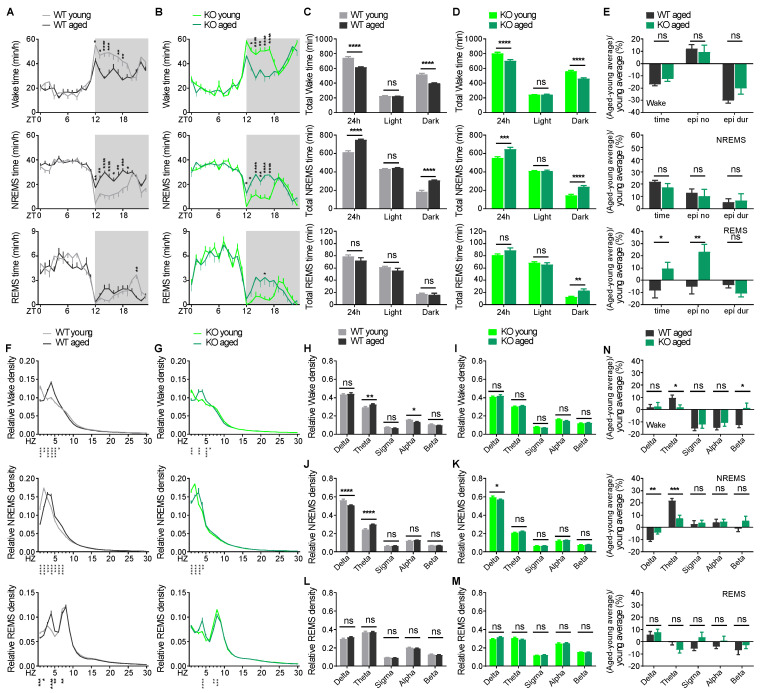
Loss of CDKL5 does not exacerbate the rate of age-associated changes in sleep behavior and EEG spectra in mice. (**A**,**B**) Hourly time in wakefulness, NREMS, and REMS in WT (young, *n* = 13; aged, *n* = 12) (**A**) and *Cdkl5* KO (young, *n* = 13; aged, *n* = 10) (**B**) mice. (**C**,**D**) Total time in wakefulness, NREMS, and REMS in WT (young, *n* = 13; aged, *n* = 12) (**C**) and *Cdkl5* KO (young, *n* = 13; aged, *n* = 10) (**D**) mice. (**E**) Normalized time, episode number, and episode duration in wakefulness, NREMS, and REMS of aged WT and KO mice. Normalized values were calculated as [(aged-young average)/young average]% in 24 h. (**F**,**G**) EEG power spectra during wakefulness, NREMS, and REMS in WT (young, *n* = 13; aged, *n* = 11) (**F**) and KO (young, *n* = 13; aged, *n* = 9) (**G**) mice. (**H**–**M**) EEG frequency bands during wakefulness, NREMS, and REMS in WT (**H**,**J**,**L**) and KO (**I**,**K**,**M**) mice. (**N**) Normalized EEG frequency band powers in NREMS, REMS, and wakefulness of aged WT and KO mice. Normalized values were calculated as [(aged-young average)/young average]% in 24 h. Data are mean ± SEM. Two-way repeated measures ANOVA with Sidak’s test (**A**,**B**, **F**–**M**) and unpaired *t*-test (**C**–**E**,**N**). * *p* < 0.05; ** *p* < 0.01; *** *p* < 0.001; **** *p* < 0.0001. ns, not significant.

Normal aging is associated with a profound decline in sleep quality in humans and rodents, including difficulty in initiating sleep, sleep fragmentation, decreased sleep efficiency, and changes in EEG oscillations [40,41]. To evaluate whether loss of CDKL5 exacerbates the rate of age-associated sleep deterioration, we directly compared baseline sleep amount, architecture, and EEG spectral profiles in young versus aged mice of each genotype, respectively. Aged WT mice showed a comparable REMS amount but decreased wakefulness and increased NREMS relative to young WT mice, which occurred mostly during the dark phase (Figure 3A,C), as previously reported [40,41,42]. The changes of sleep–wake amount during aging in KO mice followed the same pattern as in WT mice (Figure 3B,D).

Regarding sleep architecture, the number of wake episodes over 24 h in WT and KO mice was not affected by age, whereas aged mice of both genotypes had shorter wake episode durations (Figure S3A–D). During NREMS, there was a trend of more episode numbers over 24 h in aged WT (*p* = 0.086) and KO (*p* = 0.136) mice relative to young mice of each genotype (Figure S3E,F), but no significant difference was found in the episode duration (Figure S3G,H). The number and duration of REMS episodes did not differ between young and aged WT mice (Figure S3I,K). In contrast, more and shorter REMS episodes were observed in aged KO mice relative to young KO mice (Figure S3J,L).

Because young KO mice already exhibited significant differences in sleep parameters and EEG spectra compared to WT littermates (Figure 1 and Figure 2), to account for this, we normalized the time, episode number, and duration of aged mice to young mice (average group value) for each vigilance state and each genotype. Statistical analysis of the normalized sleep parameters showed that in wakefulness and NREMS, the percentage change of wake and NREMS amount, episode number, and duration during aging did not differ between WT and KO mice. In REMS, WT and KO mice, during aging, had a significant opposite change in REMS amount and episode number and a similar change in episode duration (Figure 3E). These results suggest that KO mice mainly experience a natural physiological change of sleep amount and architecture as in WT mice during aging except for an increase in REMS amount and episode number.

To study the changes of EEG parameters in WT and KO mice during aging, we calculated the spectral distribution of EEG power densities (Figure 3F,G). During wakefulness, aged WT mice exhibited increased theta power and decreased alpha power compared to young mice, while aged KO mice showed no difference in all EEG frequency bands relative to young KO mice (Figure 3H,I). During NREMS, both aged WT and KO mice showed reduced delta power relative to young WT and KO mice, respectively. Aged WT mice also had higher theta power relative to young WT mice (Figure 3J,K). The REMS EEG spectra in aged WT and KO mice were similar to young WT or KO mice, respectively (Figure 3L,M). We normalized EEG frequency band power of aged mice to the young mice (average group value) for each vigilance state and each genotype. The analysis of the normalized frequency band power showed that the percentage change in EEG power spectra during aging did not differ between genotypes, except that aged WT mice had a significant increase in normalized theta and a decrease in beta power in wakefulness, and a decrease in normalized delta and an increase in theta power in NREMS, compared to aged KO mice (Figure 3N).

### 2.4. Cdkl5 KO Mice Exhibited Normal Homeostatic Sleep Response and Circadian Rhythm

**Figure 4:**
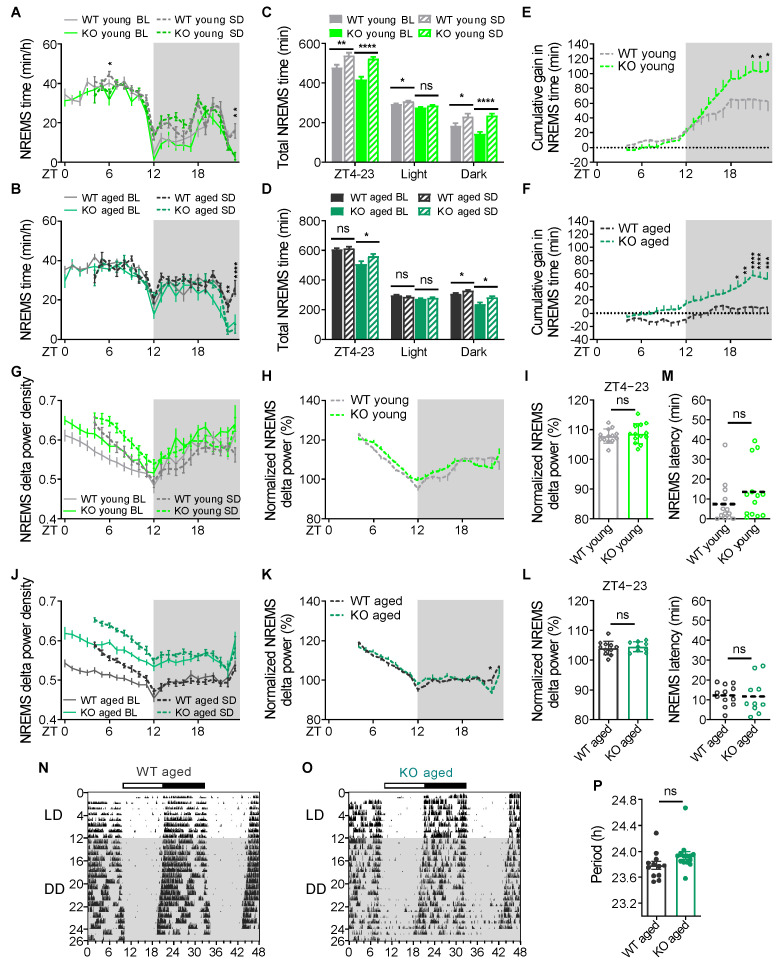
*Cdkl5* KO mice exhibited a normal homeostatic sleep response and circadian rhythm. (**A**,**B**) Hourly NREMS time of young (WT, *n* = 13; KO, *n* = 13) (**A**) and aged (WT, *n* = 12; KO, *n* = 10) (**B**) WT and *Cdkl5* KO mice before (baseline, BL) and after sleep deprivation (SD). (**C**,**D**) Amount of NREMS in young (**C**) and aged (**D**) WT and KO mice during 20 h recovery period and time-matched baseline period. (**E**,**F**) Time course of cumulative NREMS gain in young (**E**) and aged (**F**) WT and KO mice across 20 h recovery period. (**G**) Hourly NREMS delta power density of young WT and KO mice before and after sleep deprivation. (**H**,**I**) Normalized hourly (**H**) and mean (**I**) NREM delta power of young WT and KO mice during 20 h recovery period after sleep deprivation. (**J**) Hourly NREMS delta power density of aged WT and KO mice before and after sleep deprivation. (**K**,**L**) Normalized hourly (**K**) and mean (**L**) NREM delta power of aged WT and KO mice during 20 h recovery period after sleep deprivation. (**M**) Sleep latency after sleep deprivation in young (up) and aged (down) WT and KO mice. (**N**,**P**) Representative double-plotted actograms of an aged WT (**N**) and KO (**O**) mice under LD and DD conditions. (**P**) Average circadian free-running periods in DD in aged WT (*n* = 12) and KO (*n* =14) mice. Data are the mean ± SEM. Two-way repeated measures ANOVA with Sidak’s test (**A**,**B**,**E**,**F**,**H**,**K**), paired *t*-test (**C**–**D**), and unpaired t-test (**I**,**L**,**M**,**P**). * *p* < 0.05; ** *p* < 0.01; *** *p* < 0.001; **** *p* < 0.0001. ns, not significant.

According to the “two-process” model of sleep regulation, sleep is regulated by homeostatic and circadian processes [43,44]. To assess the homeostatic modulation of sleep in KO mice, we sleep-deprived KO and WT mice for 4 h, starting at the onset of the light phase. After sleep deprivation (SD), KO and WT mice exhibited similar latency to enter NREMS at both ages (Figure 4M). Young and aged animals of the KO and WT groups showed homeostatic responses to SD, with increased NREMS and REMS and decreased wakefulness relative to time-matched baseline. This effect was most pronounced in the dark phase, possibly owing to a ceiling effect in the light phase (Figure 4A–D, Figure S4A,B,D,E). We calculated the cumulative gains in NREMS, REMS, and wakefulness from the corresponding baseline. KO mice gained more NREMS and lost more wakefulness compared to WT littermates at both ages by the end of recovery period. Young KO mice also gained more REMS (Figure 4E,F and Figure S4C,F). Changes in NREM EEG delta power were similar between KO and WT mice all over the recovery period at both ages, suggesting that KO mice accumulated sleep need similarly to WT mice (Figure 4G–L). These results suggest that homeostatic responses to SD in KO mice are intact. KO mice gained more recovery sleep, possibly due to their significantly reduced baseline sleep.

After SD, young and aged KO mice had a significant increase in the episode number of wake and NREMS (Figure S4G,H) and a shorter mean episode duration of NREMS compared to WT littermates over recovery period (Figure S4I,J). Aged KO mice also had more episodes of REMS (Figure S4H). Consistently, there were more transitions between NREMS and wakefulness in KO mice (Figure S4K). Moreover, there was a significant decrease in NREMS and a concomitant increase in wakefulness at ZT23 in young KO mice and at ZT22–23 in aged KO mice at the end of the recovery phase, respectively (Figure 4A,B and Figure S4A,D). These results were consistent with the trend observed in the baseline data, suggesting that after SD, KO mice also had fragmented recovery sleep and a nightly pre-sleep arousal before the light phase compared to WT littermates.

We then examined the circadian rhythm of aged KO mice by measuring wheel-running activity. There was no difference between KO and WT mice for circadian period length during the constant darkness (Figure 4N–P). These results demonstrate that the sleep disturbances in KO mice do not result from either an impaired homeostatic or circadian regulation of sleep.

### 2.5. Selective Loss of Cdkl5 in Glutamatergic Neurons Results in Sleep Disturbances

Previous studies have shown that selective ablation of *Cdkl5* in glutamatergic and GABAergic neurons led to distinct behavioral deficits in mice [45,46]. To investigate the neuron type in which CDKL5 deficiency causes sleep disturbances, we generated conditional knockout (cKO) male mice lacking CDKL5 in glutamatergic neurons (*Vglut2^cre/+^; Cdkl5^flox/Y^* mice, hereon referred to as Vglut2-cKO) or GABAergic neurons (*Vgat^cre/+^; Cdkl5^flox/Y^* mice, hereon referred to as Vgat-cKO) by crossing *Cdkl5^flox^* female mice with *Vglut2^cre^* or *Vgat^cre^* male mice, respectively (Figure S5).

**Figure 5:**
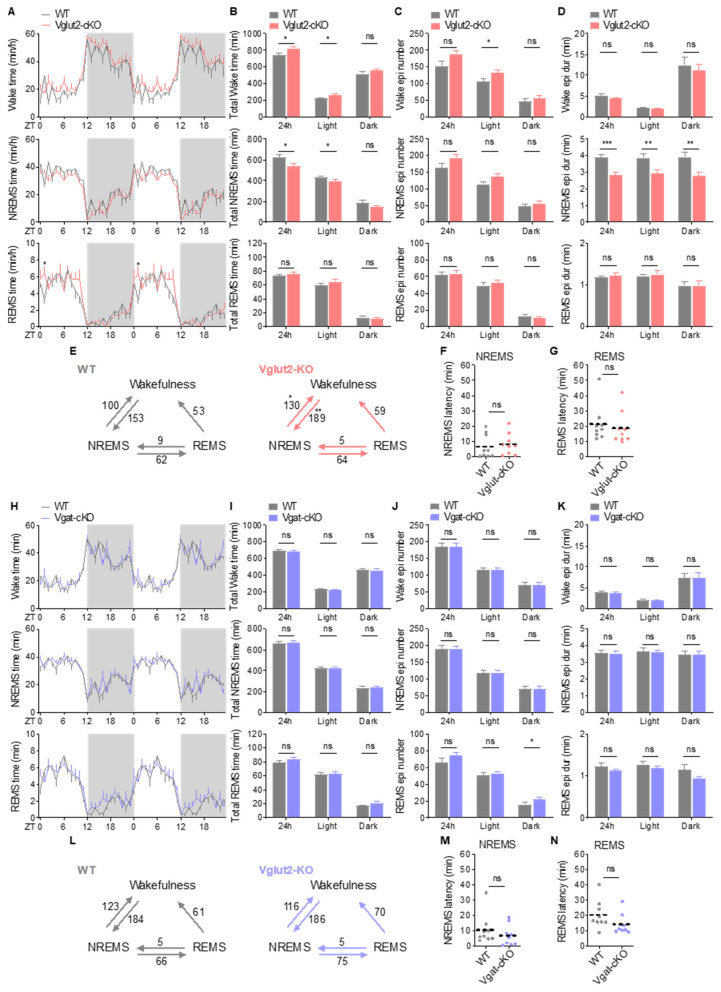
Selective loss of CDKL5 in glutamatergic neurons results in sleep disturbances. (**A**–**D**) Hourly time (**A**), total time (**B**), numbers of episodes (**C**), and mean durations of episodes (**D**) of wakefulness, NREMS, and REMS in WT (*n* = 7) and Vglut2-cKO mice (*n* = 9). Time points are double-plotted to facilitate visual detection of daily variation. (**E**) Number of transitions between wakefulness, NREMS, and REMS per 24 h in WT and Vglut2-cKO mice. (**F**,**G**) Mean NREMS (**F**) and REMS (**G**) latency in WT (*n* = 9) and Vglut2-cKO (*n* = 9) mice. (**H**,**K**) Hourly time (**H**), total time (**I**), numbers of episodes (**J**), and mean durations of episodes (**K**) of wakefulness, NREMS, and REMS in WT (*n* = 9) and Vgat-cKO mice (*n* = 9). Time points are double-plotted to facilitate visual detection of daily variation. (**L**) Number of transitions between wakefulness, NREMS, and REMS per 24 h in WT and Vgat-cKO mice. (**M**,**N**) Mean NREMS (**M**) and REMS (**N**) latency in WT (*n* = 9) and Vgat-cKO (*n* = 9) mice. Data are the mean ± SEM. Two-way repeated measures ANOVA with Sidak’s test (A,E,H,L) and unpaired t-test (**B**–**D**,**F**,**G**,**I**–**K**,**M**,**N**). * *p* < 0.05; ** *p* < 0.01; *** *p* < 0.001; ns, not significant.

Similarly to constitutive KO mice, Vglut2-cKO mice exhibited increased duration of wakefulness, decreased NREMS amount, and comparable REMS amount relative to WT littermates (Figure 5A,B). The reduction in NREMS amount in Vglut2-cKO mice occurred across the 24 h cycle (Figure 5A). Vglut2-cKO mice showed a trend of increased episode number of wakefulness (*p* = 0.0539) and NREMS (*p* = 0.0786) (Figure 5C), whereas the average episode duration of NREMS was shorter in Vglut2-cKO mice than in WT littermates (Figure 5D). Consistently, Vglut2-cKO mice, like KO mice, showed increased transitions between NREMS and wakefulness relative to WT littermates (Figure 5E). These results indicate that Vglut2-cKO mutants replicate the sleep reduction and sleep fragmentation phenotypes observed in age-matched young KO mice. However, Vglut2-cKO mice did not show increased wakefulness and decreased NREMS amount before the light phase (Figure 5A) and had comparable NREMS and REMS latencies (Figure 5F,G) relative to WT littermates, suggesting that Vglut2-cKO mice did not experience pre-sleep arousal and difficulty in initiating sleep. In contrast to Vglut2-cKO mice, Vgat-cKO animals exhibited sleep patterns similar to those observed in WT littermates. There were no significant differences in sleep–wake amount, sleep architectures, transition between vigilance states, and NREMS and REMS latencies between two groups (Figure 5H–N).

**Figure 6:**
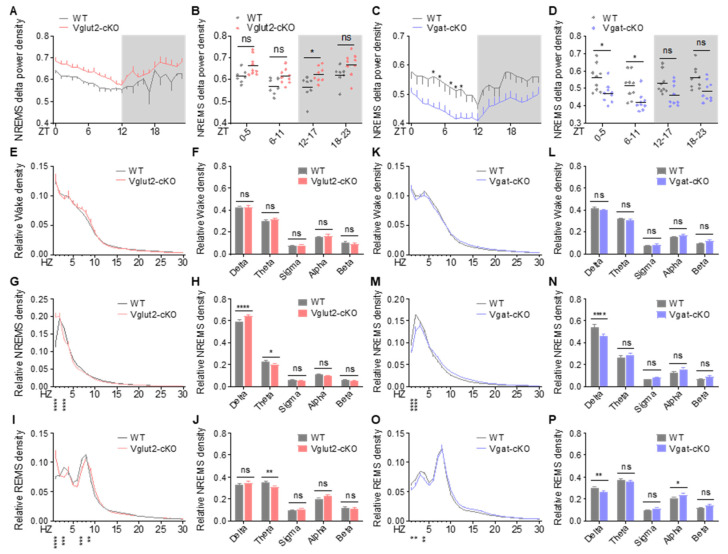
Alterations in EEG spectral power in Vglut2-cKO and Vgat-cKO mice. (**A**,**B**) Mean NREMS delta power in every hour (**A**) and per 6 h block (**B**) in WT (*n* = 7) and Vglut2-cKO mice (*n* = 9). (**C**,**D**) Mean NREMS delta power in every hour (**C**) and per 6 h block (**D**) in WT (*n* = 9) and Vgat-cKO mice (*n* = 9). (**E**–**J**) EEG power spectra and frequency bands during wakefulness (**E**,**F**), NREMS (**G**,**H**), and REMS (**I**,**J**) in WT and Vglut2-cKO mice. (**K**–**P**) EEG power spectra and frequency bands during wakefulness (**K**,**L**), NREMS (**M**,**N**), and REMS (**O**,**P**) in WT and Vgat-cKO mice. Data are the mean ± SEM. Two-way repeated measures ANOVA with Sidak’s test (**A**–**P**). * *p* < 0.05; ** *p* < 0.01; *** *p* < 0.001; **** *p* < 0.0001. ns, not significant.

We next examined baseline EEG power spectra in cKO mice. Loss of CDKL5 in glutamatergic and GABAergic neurons resulted in distinct EEG spectral changes in mice. Specifically, Vglut2-cKO mice exhibited elevated NREMS delta power across the 24 h cycle (Figure 6A,B). During wakefulness, Vglut2-cKO had similar EEG power spectral density profiles to WT littermates (Figure 6E,F). During NREMS, Vglut2-cKO mice, like constitutive KO animals, had an increase in delta power and a decrease in theta power relative to WT littermates (Figure 6G,H). During REMS, Vglut2-cKO mice showed decreased theta power and a trend toward increased alpha power (*p* = 0.0907). We did not find an increase in beta power in Vglut2-cKO mice as in KO animals (Figure 6I,J). These data suggest that the alterations of EEG spectra in Vglut2-cKO mice largely parallelled constitutive KO mouse EEG spectral changes relative to WT mice. In contrast to Vglut2-cKO mice, Vgat-cKO had significantly lower EEG delta power during NREMS across the 24 h cycle than WT littermates (Figure 6C,D). Power spectral density analysis showed comparable EEG spectra in all vigilance states between Vgat-cKO and WT littermates, except that Vgat-cKO mice had decreased delta power in NREMS and REMS and increased alpha power in REMS (Figure 6K,P). Taken together, our results indicate that loss of CDKL5 in excitatory neurons, but not inhibitory neurons, leads to sleep disturbances.

To assess the integrity of the sleep homeostat in Vglut2-cKO mice, we sleep-deprived mice for 4 h starting at the onset of the light phase. After SD, Vglut2-cKO and WT littermate mice showed similar latency to enter NREMS (Figure S6G). Vglut2-cKO mice exhibited a significant rebound in NREMS and REMS and a decrease in wake time relative to the time-matched baseline (Figure S6A,B). The cumulative gains in NREMS, REMS, and wakefulness from corresponding baseline values did not differ between Vglut2-cKO and WT littermates by the end of the 20 h recovery period (Figure S6C). Changes in NREM EEG delta power were also similar between two genotypes across the recovery period (Figure S6D–F). Collectively, these data suggest that Vglut2-cKO mice, like constitutive KO mice, have an unimpaired homeostatic response to sleep deprivation.

## 3. Discussion

This study is the first to comprehensively analyze sleep phenotypes and sleep EEG activity in mouse models of CDD using EEG/EMG recording. The absence of spontaneous seizures in *Cdkl5* KO, Vglut2-cKO, and Vgat-cKO mice allowed us to identify the alterations of sleep and EEG power spectra solely attributed to the loss of CDKL5 activity rather than the secondary consequence of seizures. Patients with CDD suffer sleep disturbances, including difficulty falling and staying asleep, frequent night awakenings, shorter total sleep time, and decreased REMS. We found that KO mice mirror all the sleep phenotypes in CDD patients except for the REMS amount (Figure 1). In addition, we identified pre-sleep arousal in KO mice that has also been observed in autism spectrum disorder (ASD) patients [47]. Relative to WT littermates KO mice had comparable REMS at a young age and increased REMS at middle-age in contrast to reduced REMS in CDD patients. Chronic sleep deprivation could lead to depression [48,49], and increased REMS amount has been considered as a biological marker of depression [50]. Therefore, the enhanced REMS in aged KO mice might be associated with possible depression induced by chronic sleep loss. For the reduced REMS in CDD patients, we reason that it is likely attributed to the secondary consequence of seizures given that epilepsy has been shown to suppress REMS in humans and animals [51,52,53,54].

The characterization of sleep EEG power spectra in CDD patients has not been reported. In KO mice, we identified similar EEG power alterations compared to WT littermates at both ages. Specifically, KO mice exhibited elevated NREMS delta power which is consistent with findings in RTT and AS patients [22,24]. Increased NREMS delta power indicates a hyper-synchronization of neuronal firing [36]. Given that the hypersynchrony and hyperexcitability of neuronal networks link to the generation of epileptic activity [55,56], the elevated NREMS delta activity that is more prominent with age in *Cdkl5* KO mice (Figure 2) might account for the enhanced seizure susceptibility to kindling in KO mice and spontaneous seizures in aged female *Cdkl5* mutant mice [30,57,58,59]. KO mice had reduced theta activity in both NREMS and REMS, and enhanced alpha and beta activity in REMS. Theta waves in sleep play a role in processing information and memory consolidation [60,61]. The compromised theta power in NREMS and REMS is consistent with the marked cognitive deficits in KO mice. Alpha and beta activities during sleep reflect arousal processes [62,63]. Therefore, increased alpha and beta power in REMS sleep suggests a hyperarousal in KO mice. Taken together, our study reveals a strong face validity of KO mice in recapitulating sleep disturbances experienced by CDD patients and establishes a causality link between *Cdkl5* loss-of-function and alterations in sleep and EEG spectra independent of secondary consequences of seizures in KO mice.

Aging in healthy individuals is associated with a profound decline of sleep quality and EEG activity [40,41,42]. Direct comparisons between young and aged mice of each genotype revealed that KO and WT mice underwent similar age-related changes in the temporal distribution of wake and sleep, sleep amount and architecture, and EEG spectral power (Figure 3A–D,F–I). Moreover, KO and WT mice exhibited comparable rates of age-associated sleep and EEG deterioration except for a few sleep and EEG parameters. The positive gain in REMS in aged KO mice, in contrast to negative gain in aged WT mice relative to young mice, may be modulated by possible depression in aged KO mice, as we discussed earlier (Figure 3E). Aged KO and WT mice showed a decreased NREMS delta power relative to young animals (Figure 3J,K), which is consistent with the findings in human data [64]. The reduction ratio of NREMS delta power is significantly smaller in aged KO mice compared to aged WT mice. This might be due to the implication of hypersynchrony of neuronal activity attributed to *Cdkl5* loss-of-function. Aged WT mice showed a significant increase in NREMS theta gain, possibly because their NREMS delta power was largely reduced (Figure 3N). Taken together, our natural history study indicates that KO mice during aging mainly experience a physiological decline in sleep architecture and EEG activity at the same rate to WT mice, and the loss of CDKL5 does not exacerbate the rate of age-related sleep deterioration. This suggests that the mechanisms underlying the sleep disturbances in CDD might not alter with age, and mechanism-based therapies for improving sleep quality would be effective for patients across all age groups.

Sleep is regulated by homeostatic and circadian processes, and dysfunction of either one or both of these systems may underlie the sleep disturbances in CDD. KO mice had intact homeostatic responses to SD and showed no gross abnormalities in circadian rhythms, pointing to sleep disturbances in KO mice not being attributed to impaired homeostatic and circadian regulation. Further, KO mice exhibited higher baseline NREMS delta power, indicating that an intrinsic decrease in sleep need is not the pathophysiological mechanism behind insomnia in KO mice. Genetic manipulation of *Cdkl5* expression in glutamatergic and GABAergic neurons revealed that Vglut2-cKO mice replicated alterations of sleep and EEG parameters in constitutive KO mice except for the absence of pre-sleep arousal, prolonged sleep onset latency, and enhanced alpha and beta power in REMS. On the contrasty, Vgat-cKO mice exhibited comparable sleep phenotypes and EEG spectra relative to WT littermates except for reduced delta power in NREMS and REMS. These results uncover the cellular origin of CDD-related sleep disturbances; that is, disorders of maintaining sleep, frequent night awakenings, and shorter total sleep times are accounted for by the loss of CDKL5 in excitatory neurons. Interestingly, pre-sleep arousal and prolonged sleep onset latency were not found in either Vglut2-cKO or Vgat-cKO mice. These phenotypes may be mediated by CDKL5 function in other cellular origins, including peripheral tissues, or by the synergistic functions of CDKL5 in glutamatergic and GABAergic neurons. Vgat-cKO mice showed diminished delta power in NREMS and REMS. While the factors for this network desynchronization are unclear, it may suggest that the neural network is more hyper-synchronized in mice selectively lacking CDKL5 in excitatory neurons than in constitutive KO mice. This could explain that aged heterozygous *Cdkl5* mutant mice exhibited only spontaneous epileptic spasms, while mice with specific deletion of *Cdkl5* in excitatory neurons exhibited severe tonic–clonic seizures [57,59,65].

CDKL5 interacts with and phosphorylates HDAC4 [66]. Recent studies reveal that HDAC4 is a sleep-regulating molecule, acting as a substrate for salt-inducible kinase 3 (SIK3) to regulate daily sleep amount in mice [67,68]. In the absence of SIK3, hypophosphorylated HDAC4 translocates to the nucleus, which leads to insomniac phenotypes by repressing the transcription of sleep-promoting genes. Intriguingly, conditional knockout of Sik3 in excitatory but not inhibitory neurons results in reduced total sleep time and sleep fragmentation similar to that in CDKL5 Vglut2-cKO mice [68]. Given the broad expression of SIK3, HDAC4, and CDKL5 in the brain and the analogous CDKL5-dependent HDAC4 subcellular localization [66,67,69], we speculate that the nuclear retention of hypophosphorylated HDAC4, due to *Cdkl5* loss-of-function, might contribute to sleep disturbances in KO mice. HDAC4 inhibitor treatment has shown to decrease HDAC4 nuclear accumulation in neuronal precursor cells of *Cdkl5* KO mice [66]. Further studies are needed to test our hypothesis by examining whether HDAC4 inhibitor treatment can increase the sleep amount and rescue sleep fragmentation in KO mice.

Developing reliable, reproducible, and translatable biomarkers to facilitate the success of clinical trials for CDD is an urgent need. Sleep and sleep EEG parameters are objective measures, translatable between animal models and humans and stable from night to night in the same individual [70]. Enhanced sleep delta power and reduced REMS amount have shown to be potential biomarkers for some neurodevelopmental disorders and epilepsy [22,24,54]. Early onset refractory epilepsy is one of the hallmark features of CDD. Seizures can adversely affect quality and quantity of sleep [71,72] and EEG patterns [17]. In current study, KO mice exhibited higher NREMS delta power and various alterations in sleep parameters, EEG features, and EEG band ratios compared with WT littermates, which were similar across ages. Given the absence of seizures in our KO mice, these data provide valuable information and a firm foundation to facilitate the search for sensitive biomarkers to monitor disease progression, including seizures, and evaluate the efficacy of novel therapeutics in CDD. Future work comparing the natural history data from this study with sleep and EEG data from CDD patients and mice with spontaneous and recurrent seizures will help to identify sensitive and translatable biomarkers for preclinical and clinical trials.

The limitation of this study was that only male mice were used. Given that nearly 90% of patients with CDD are females, and sex-specific sleep behaviors have been reported in mice [73,74], it is necessary to extend sleep analysis to heterozygous *Cdkl5* knockout female mice. Future sleep studies using *Cdkl5* mutant female mice could lead to more accurate drug development and translational recommendations for sleep treatment in CDD.

In summary, our findings reveal that KO mice recapitulate sleep disturbances of CDD patients, and *Cdkl5* loss-of-function does not worsen the rate of age-associated sleep and EEG deteriorations. KO mice exhibited pre-sleep arousal and enhanced EEG alpha and beta activities during sleep, which might account, at least partly, for sleep disturbances in CDD, as has been reported [47,62,63]. Moreover, CDKL5 is known to directly phosphorylate HDAC4 and *Cdkl5* loss-of-function leads to HDAC4 hypophosphorylation, nuclear translocation and activation [66]. Hypophosphorylation of HDAC4 in excitatory neurons has been linked to reduction of daily NREMS amount and shorter NREMS episode duration in mice [68]. Similarly, our results show that selective loss of CDKL5 in excitatory neurons results in reduced NREMS amount and sleep fragmentation. Thus, future studies are needed to investigate whether hypophosphorylation and activation of HDAC4 might account for the sleep phenotypes in CDKL5 KO mice and whether treatment of HDAC4 inhibitors can alleviate the sleep phenotypes of CDKL5 KO mice. Together, our study provides insights into developing potential sleep treatment strategies of CDD. First, HDAC4 inhibitors may be an effective pharmacological sleep treatment for CDD patients. Second, combined medication, rather than monotherapy, is likely to enhance treatment efficacy given that more than one factor account for the sleep disturbances in KO mice as discussed above. Third, the same mechanism-based therapy could be used for CDD patients of all ages since loss of CDKL5 does not exacerbate age-associated sleep deteriorations, implying that the mechanisms underlying the sleep disturbances in CDD may not alter with age. Finally, comparing our findings with sleep and EEG data across different CDD rodent models and human patients will help to identify convergent translational biomarkers for CDD.

## 4. Materials and Methods

### 4.1. Mice

Animal experimental procedures were approved and performed in accordance with the Institutional Animal Care and Use Committee of the University of Tsukuba and Tokyo Metropolitan Institute of Medical Science. *Cdkl5* KO (*Cdkl5^tm1.1Teta^*) and *Cdkl5^flox^* (*Cdkl5^tm1Teta^*) mouse lines on a C57BL/6N background were as previously described [33,58]. *Vglut2^cre^* (*Vglut2-ires-cre*) mice and *Vgat^cre^* (*Vgat-ires-cre*) mice maintained on a C57BL/6N background were used. Wild-type C57BL/6N (C57BL/6NJcl) mice were purchased from CLEA Japan. All mice were housed under humidity- and temperature (22 °C)-controlled conditions on a 12 h light/dark cycle with food and water ad libitum. For producing *Cdkl5* cKO mice, female mice (genotype: *Cdkl5^flox/flox^* and *Cdkl5^flox/+^*) were mated with male mice (genotype: *Vglut2^cre/+^* or genotype: *Vgat^cre/+^*, respectively). Control animals of *Cdkl5^-/Y^* (referred to as KO), *Vglut2^cre/+^*; Cdkl5^flox/Y^ (referred to as Vglut2-cKO), and *Vgat^cre/+^*; *Cdkl5^flox/Y^* (referred to as Vgat-cKO) mice were age- and sex-matched wild-type littermates.

Given the confounding effects of mosaic CDKL5 expression in females due to random X-chromosome inactivation, and given that the majority of patients with CDD are younger than 45 years [3,75], we used male young adult *Cdkl5* KO mice (3-month-old), which have been used in most reported studies [34,35,58], and middle-aged KO mice (12-months olds, corresponding to 45 years in humans) [75]. For *Cdkl5* cKO mice, 3-month-old Vglut2-cKO and Vgat-cKO and their WT littermates were used.

### 4.2. EEG/EMG Electrode Implantation

At 3 months or 12 months of age, male mice were stereotaxically implanted with EEG/ EMG electrodes under isoflurane anesthesia (4% for induction and 2% for maintenance). Mice were implanted with an electrode assembly with 4 EEG electrode pins and 2 flexible stainless EMG electrode wires. After the coordinate of lambda point was set as (0, 0, 0), 4 EEG electrode pins were placed over the frontal and occipital cortices (anteroposterior (AP): 0.5 mm; mediolateral (ML): ±1.3 mm; dorsoventral (DV): −1.3 mm and AP: −4.5 mm; ML: ±1.3 mm, DV: −1.3 mm) under stereotaxic control and subsequently fixed to the skull using dental cement (3M ESPE, RelyX U200 Unicem Syringe Dental Resin Cement, 3M Company, St. Paul, MN, USA). The EMG wires were bilaterally placed into both trapezius muscles. The mice were singly housed and allowed to recover from surgery for at least 7 days. After the recovery period, the mice were attached to a recording cable and permitted habituation in the recording environment for at least one week before sleep recording.

### 4.3. EEG/EMG Data Acquisition and Analysis

After habituation, mice were subjected to a 24 h undisturbed baseline EEG and EMG recording starting at the onset of light phase (ZT0). The following day, the mice were sleep-deprived on an automated orbital shaker for 4 h from ZT0–ZT3 under the same humidity, temperature, and lighting conditions as those in the normal breeding environment [76]. EEG and EMG data were collected and analyzed as described before with some modifications [76,77]. Briefly, EEG/EMG signals were amplified with a multichannel amplifier (NIHON KODEN, #AB-611J, Nihon Kohden Corporation, Tokyo, Japan) and digitized at a sampling rate of 250 Hz using an analog-to-digital converter (National Instruments #PCI-6220). EEG and EMG were high- and low-pass band filtered (EEG: 0.5–100 Hz; EMG: 5–300 Hz).

EEG/EMG recording data were analyzed using a MATLAB Version 7.16-based semi-automated staging software followed by manual correction. EEG signals were decomposed by fast Fourier transform analysis for 1 to 30 Hz with 1 Hz bins, which was conducted in a state-dependent manner. Sleep–wake states were scored offline in 20 s epochs based on EEG patterns of delta power (1–4 Hz), theta power (5–8 Hz), and EMG signals. Each epoch was classified as wake (low amplitude, fast EEG and high amplitude, variable EMG), non-rapid eye movement sleep (NREMS) (high amplitude delta power EEG and low EMG tonus), and REMS (theta-dominate EEG and very low amplitude EMG representing atonia). Hourly plots of NREMS/REMS/wake time were calculated by summing up all 20 s epochs scored to be NREMS/REMS/wake per hour. Baseline NREMS latency was defined as the time from the onset of the light phase to the appearance of the first solid NREMS episode (>20 s). Baseline REMS latency was defined as the time between the onset of the first episode of NREMS and the first episode of solid REMS (>20 s). The sleep latency after sleep deprivation was defined as the time elapsed from the end of deprivation to the onset of the first episode of solid NREMS.

Relative EEG power density is defined as the ratio of a specific frequency bin to the total state-specific power over all frequency bins (1–30 Hz). EEG power spectral density was analyzed for 1 Hz bins and standard frequency bands (delta: 1–4 Hz; theta: 5–8 Hz; sigma: 10–12 Hz; alpha: 9–14 Hz; and beta: 15–30 Hz). Power ratios of theta/delta, alpha/delta, beta/delta, alpha/theta, beta/theta, and beta/alpha were calculated. For hourly NREMS delta power density analysis after sleep deprivation, values of delta power for each hour after sleep deprivation were normalized to the baseline average NREMS delta power from ZT8 to ZT11, which is at the end of the major rest period [78,79]. Epochs containing recording artefacts were included in the sleep–wake state totals and architecture analysis but excluded from spectral analysis. One 12-month-old WT and one *Cdkl5* KO mouse were excluded from all EEG spectral analyses due to excessive EEG artefacts. Time-locked video was used to assess the occurrence of seizure, as previously reported [57].

### 4.4. Assessment of Circadian Rhythms

Mice were placed in individual wheel running cages with free access to food and water and kept under a 12:12 h light–dark (LD) cycle (light: 200 lux) for 2 weeks followed by under 2~3 weeks of constant darkness (DD). Data collection and analysis of wheel-running activity were conducted using Clocklab Version 6 software (Actimetrics). Circadian periods were calculated by periodogram analysis using the Clocklab analysis software. Activity rhythms data for 7 days in DD were used for these analyses.

### 4.5. Western Blot

Mouse brain tissues were quickly dissected after cervical dislocation, snap-frozen in liquid nitrogen, and stored at −80 °C until use. Tissues were homogenized using a rotor-stator homogenizer (Polytron, Micro-tec Co., Ltd., Urayasu, Japan) in ice-cold lysis buffer (20 mM HEPES, pH 7.5, 100 mM NaCl, 10 mM Na4P2O7, 1.5% Triton X-100,15 mM NaF, 1× PhosSTOP (Roche Diagonostics Gmbh, Mannheim, Germany), 5 mM EDTA, 1× protease inhibitor (Roche Diagonostics Gmbh, Mannheim, Germany), and then centrifuged at 13,000× *g* at 4 °C. The supernatants were separated by SDS–PAGE and transferred to the PVDF membrane using the Trans-Blot Turbo Transfer System (BIO-RAD, Hercules, CA USA). Immunoblotting was performed according to standard protocols with the following antibodies: rabbit anti-CDKL5 (1:500, HPA002847, Sigma, St. Louis, MO USA) and rabbit anti-β-tubulin(9F3) (1:5000, 2128, Cell Signaling Technology, Danvers, MA USA). The blots were then washed and incubated with HRP-conjugated, donkey anti-rabbit IgG (1:10,000, RRID: AB_2307391, Jackson ImmunoResearch Laboratories, Wet Grove, PA USA).

### 4.6. Statistical Analysis

Statistical analyses were conducted using GraphPad Prism 8 software. Student’s *t*-tests were used for pairwise comparisons. Paired *t*-test was used for matched subjects, whereas unpaired t-test for group comparisons. Two-way repeated-measures ANOVA with Sidak’s test was used to perform group comparisons with multiple measurements (for example, genotype and time, or frequency). The number of animals or samples used in each experiment are stated in the figure legends. All data were represented as mean ± standard error of the mean (SEM), and *p* < 0.05 was considered statistically significant.

## Data Availability

All data generated or analyzed during this study are included in this article. Any additional information required to reanalyze the data reported in this paper will be made available from the corresponding author upon reasonable request.

## Supplementary Materials

The following supporting information can be downloaded at https://www.mdpi.com/article/10.3390/ijms26083754/s1: Figures S1–S6.
