# Supplementary material for: Cdkl5 Knockout Mice Recapitulate Sleep Phenotypes of CDKL5 Deficient Disorder
Source: Int J Mol Sci. 2025 Apr 16;26(8):3754. doi: 10.3390/ijms26083754 (PMC12028001; doi:10.3390/ijms26083754)
Supplement: Supplementary file 1 [file ijms-26-03754-s001.zip › ijms-3515282-supplementary.pdf]

## Supplementary Figures and legends:

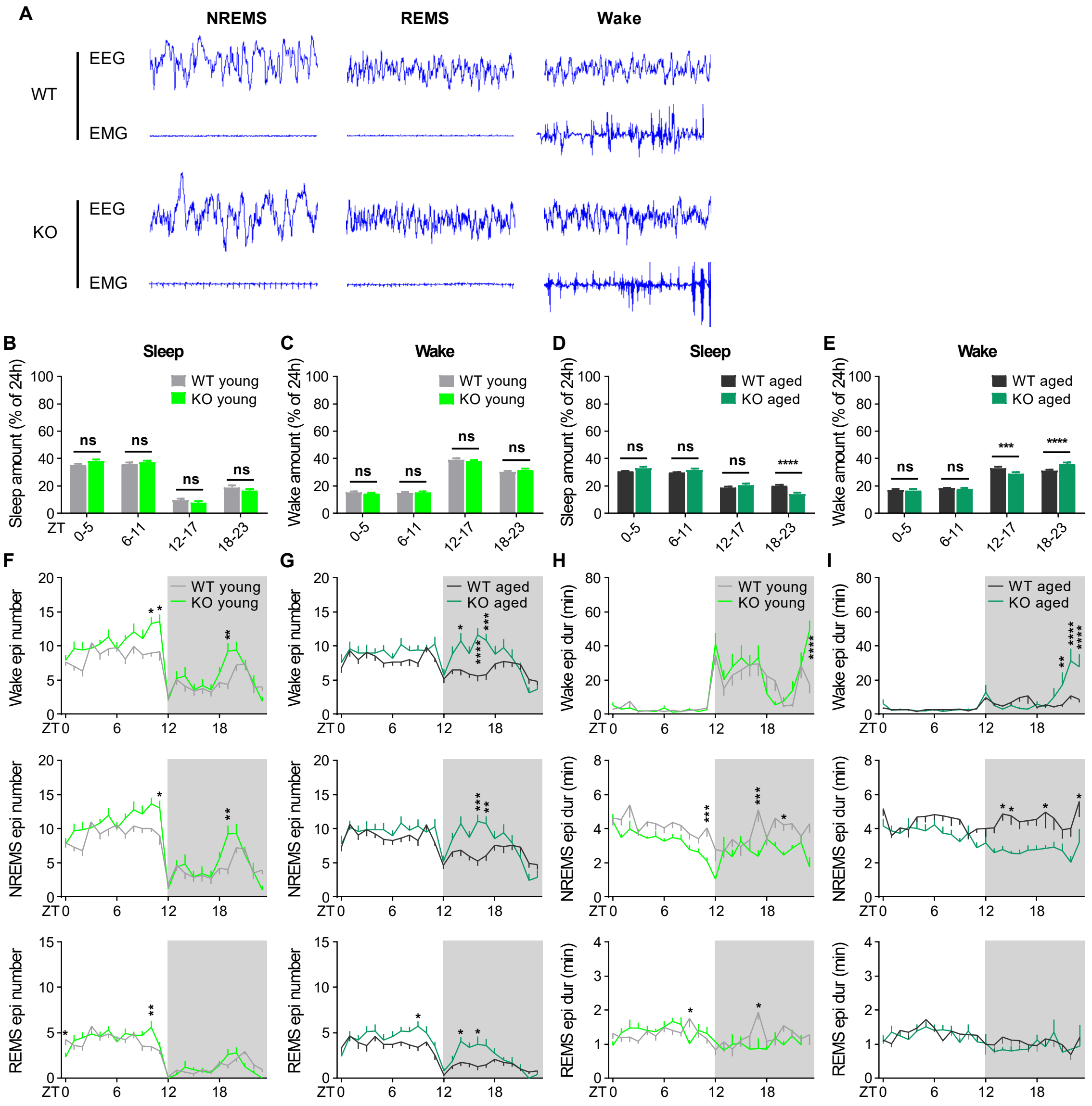


**Figure S1.** Sleep/wakefulness behaviors in *Cdkl5* KO mice. (**A**) Representative example of EEG and EMG for wakefulness, NREMS and REMS of WT and *Cdkl5* KO mice. (**B-E**) The proportion of the amount in the 6h block to the full 24h period for sleep and wakefulness in young (WT, n = 13; KO, n = 13) (**B-C**) and aged (WT, n = 12; KO, n = 10) (**D-E**) mice. (**F-G**) Hourly number of episodes of wakefulness, NREMS and REMS in young (**F**) and aged (**G**) WT and KO mice. (**H-I**) Hourly episode duration of wakefulness, NREMS and REMS in young (**H**) and aged (**I**) WT and KO mice. Data are mean ± SEM. Two-way repeated measures ANOVA with Sidak's test (B-I). *P < 0.05; **P < 0.01; ***P < 0.001; ****P < 0.0001; ns, not significant.

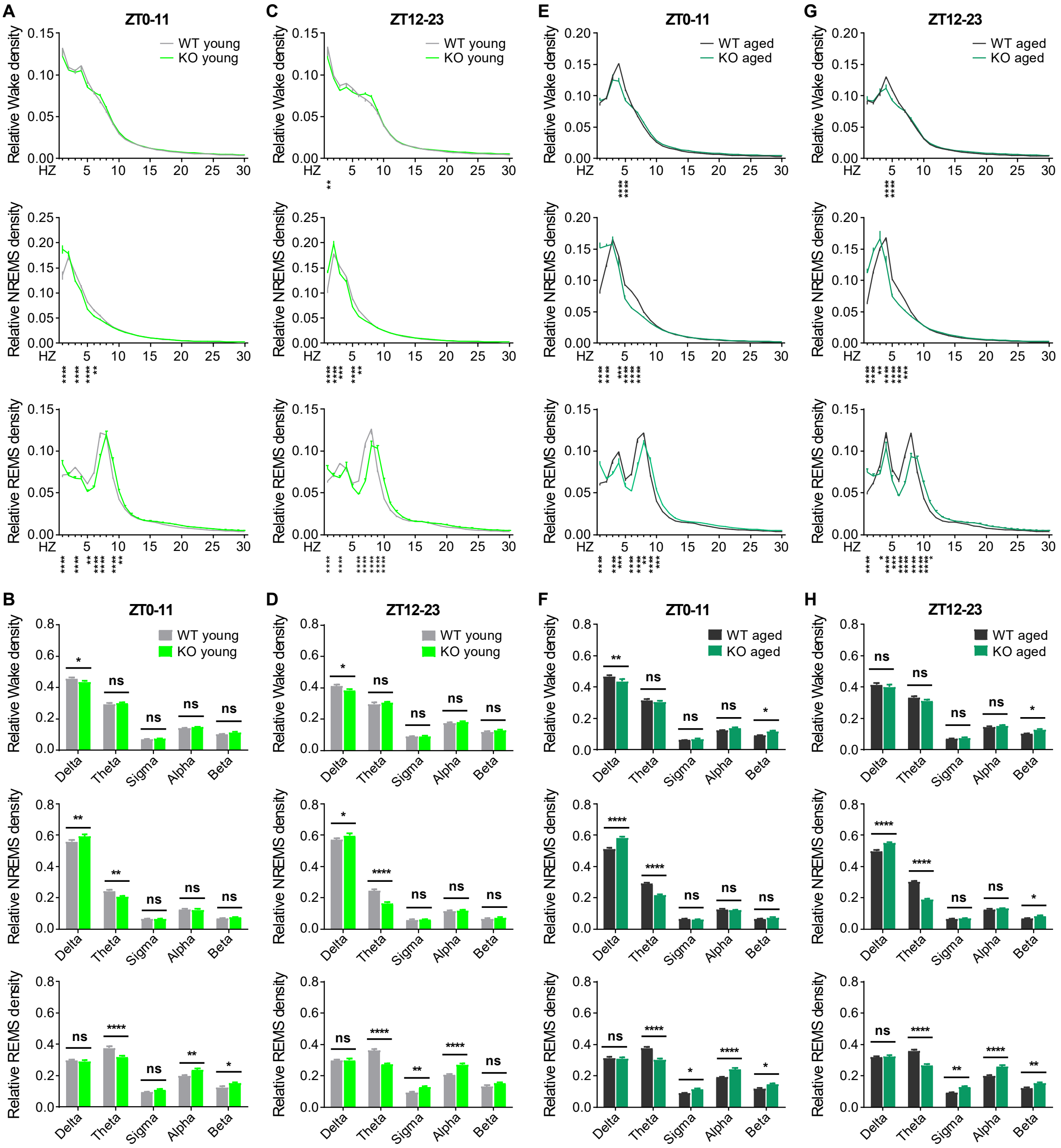


**Figure S2.** Alterations in EEG spectral power in *Cdkl5* KO mice during light and dark phases. (**A-D**) EEG power spectra and frequency bands of wakefulness, NREMS and REMS during light (**A-B**) and dark (**C-D**) phases in young WT (n = 13) and *Cdkl5* KO (n = 13) mice. (**E-H**) EEG power spectra and frequency bands of wakefulness, NREMS and REMS during light (**E-F**) and dark (**G-H**) phases in aged WT (n = 11) and KO (n = 9) mice. Data are mean ± SEM. Two-way repeated measures ANOVA with Sidak's test (A-H). *P < 0.05; **P < 0.01; ***P < 0.001; ****P < 0.0001; ns, not significant.

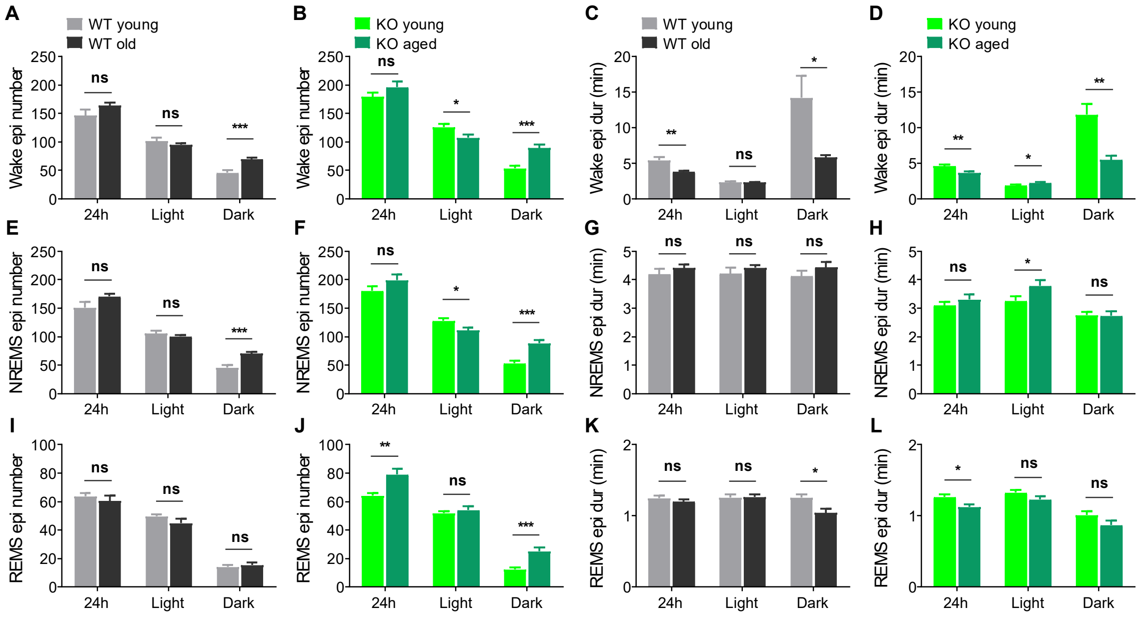


**Figure S3.** Age-dependent changes of sleep/wakefulness behaviors in *Cdkl5* KO mice. (**A-D**) Episode numbers (**A-B**) and mean durations (**C-D**) of wakefulness in young and aged WT and KO mice. (**E-H**) Episode numbers (**E-F**) and mean durations (**G-H**) of NREMS in young and aged WT and KO mice. (**I-L**) Episode numbers (**I-J**) and mean durations (**K-L**) of REMS in young and aged WT and KO mice. Data are mean ± SEM. Unpaired t-test (A-L). *P < 0.05; **P < 0.01; ***P < 0.001; ****P < 0.0001; ns, not significant.

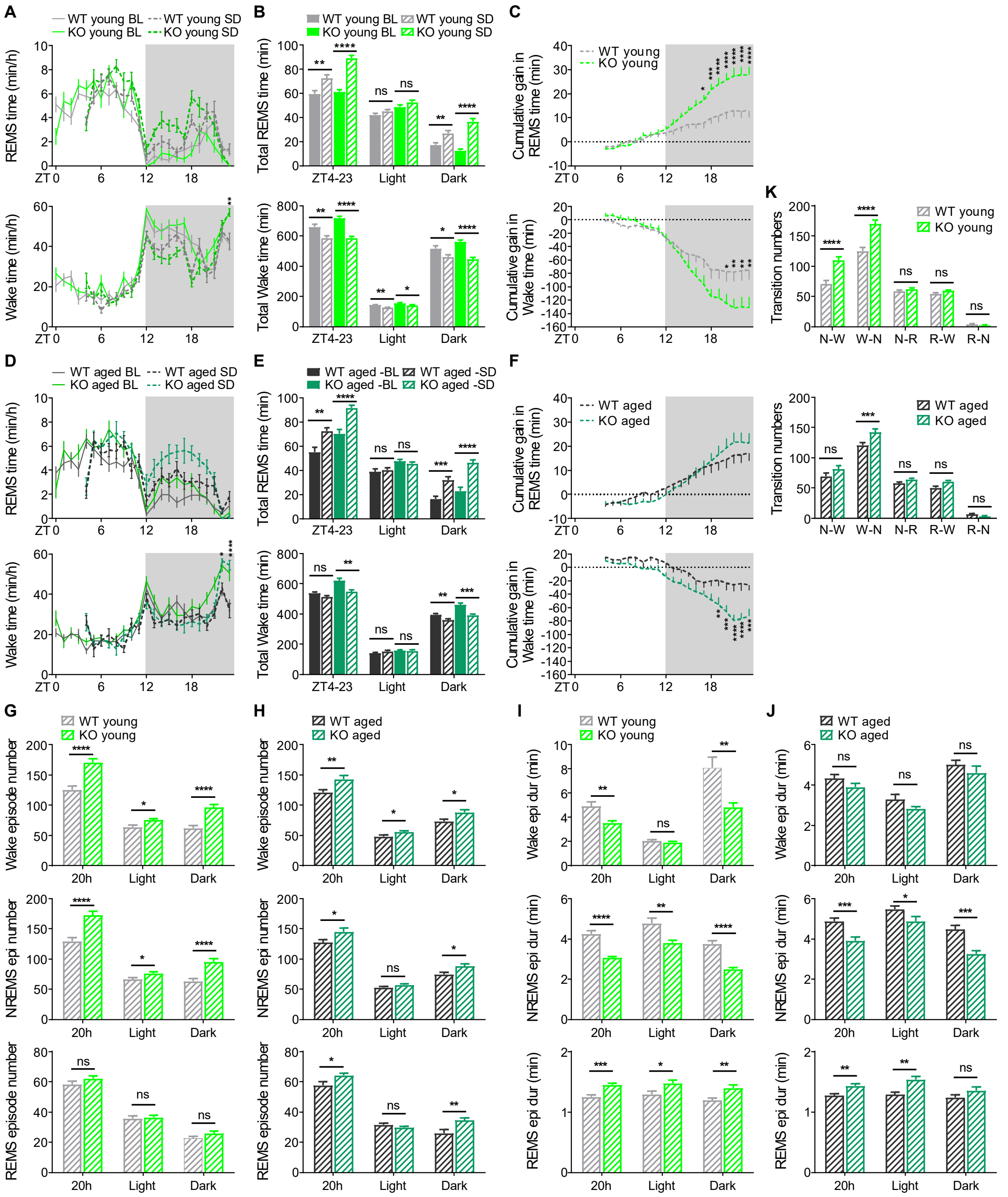


**Figure S4.** Sleep/wakefulness behaviors after sleep deprivation in *Cdkl5* KO mice. (**A**) Hourly time of REMS (up) and wakefulness (down) in young WT (n = 13) and *Cdkl5* KO mice (n = 13) before (baseline, BL) and after sleep deprivation (SD). (**B**) Amount of REMS (up) and wakefulness (down) in young WT and KO mice during 20-h recovery period and time-matched baseline period. (**C**) Time course of cumulative REMS (up) and wakefulness (down) gain in young WT and KO mice across 20-h recovery period. (**D**) Hourly time of REMS (up) and wakefulness (down) in aged WT (n = 12) and KO mice

(n = 10) before (BL) and after sleep deprivation (SD). (**E**) Amount of REMS (up) and wakefulness (down) in aged WT and KO mice during 20-h recovery period and time-matched baseline period. (**F**) Time course of cumulative REMS (up) and wakefulness (down) gain in aged WT and KO mice across 20-h recovery period. (**G-J**) Episode numbers (**G-H**) and mean durations (**I-J**) of wakefulness, NREMS and REMS in young and aged WT and KO mice. (**K**) Number of transitions between wakefulness (W), NREMS (N), and REMS (R) in young (up) and aged (down) WT and KO mice during 20-h recovery period. Data are mean ± SEM. Two-way repeated measures ANOVA with Sidak's test (A, C-D, F, K); paired t-test (B, E); and unpaired t-test (G-J). *P < 0.05; **P < 0.01; ***P < 0.001; ****P < 0.0001; ns, not significant.

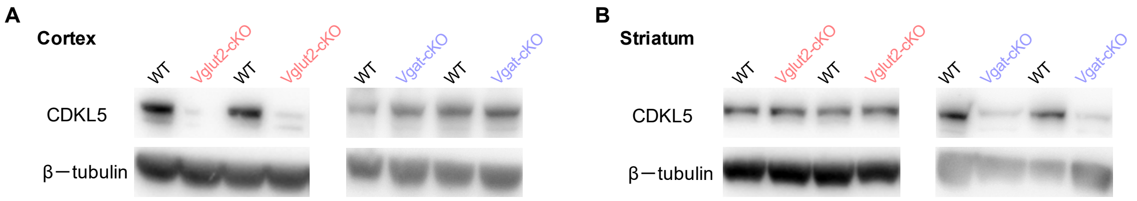


**Figure S5.** Validation of *Cdkl5* conditional knockout in Vglut2-cKO and Vgat-cKO mouse brain tissues. (**A**) In the cortex where glutamatergic neurons are enriched, Vglut2-cKO mice show reduced CDKL5 protein compared to WT littermates. **(B)** In the striatum where GABAergic neurons are enriched, Vgat-cKO mice show reduced CDKL5 protein compared to WT littermates.

**A-B**. Two pairs of WT and Vglut2-cKO, and WT and Vgat-cKO were used.

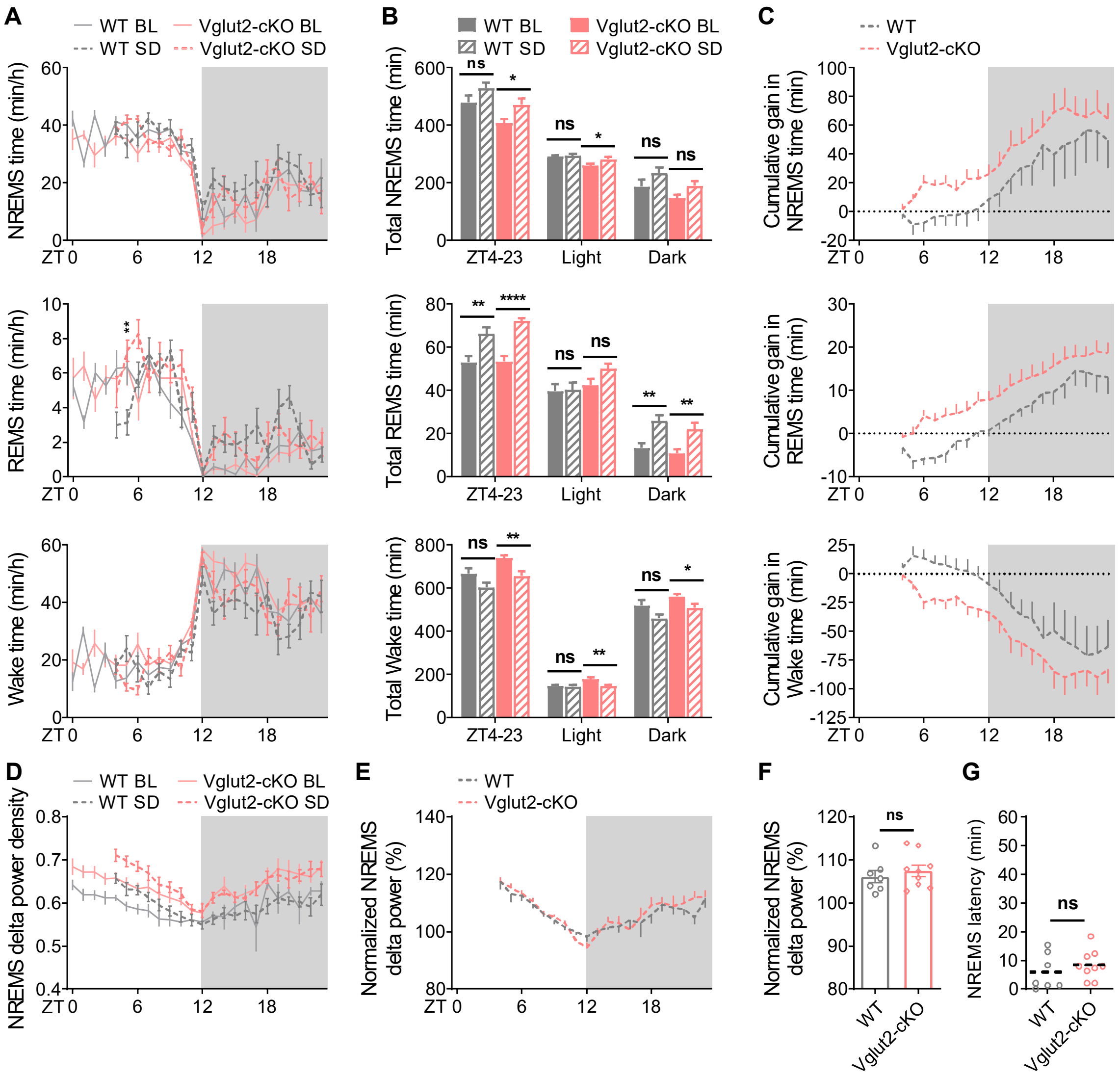


**Figure S6.** Intact homeostatic responses to sleep deprivation in Vglut2-cKO mice. (**A**) Hourly time of NREMS, REMS and wakefulness in WT (n = 7) and Vglut2-cKO mice (n = 9) before (baseline, BL) and after sleep deprivation (SD). (**B**) Amount of NREMS, REMS and wakefulness in WT and Vglut2-cKO mice during 20-h recovery period and time-matched baseline period. (**C**) Time course of cumulative NREMS, REMS and wakefulness gain in WT and Vglut2-cKO mice across 20-h recovery period. (**D**) Hourly NREMS delta power density of WT and Vglut2-cKO mice before and after sleep deprivation. (**E-F**) Normalized hourly (**E**) and mean (**F**) NREM delta power in WT and Vglut2-cKO mice during 20-h recovery period. (**G**) Sleep latency after sleep deprivation in WT and Vglut2-cKO mice. Data are mean ± SEM. Two-way repeated measures ANOVA with Sidak's test (A, C, E); paired t-test (B); and unpaired t-test (F-G). *P < 0.05; **P < 0.01; ***P < 0.001; ****P < 0.0001; ns, not significant.
